# Pain-diminishing effect of Kinesio taping in patients after sternotomy

**DOI:** 10.1186/1749-8090-10-S1-A76

**Published:** 2015-12-16

**Authors:** HM Klein, R Brockmann, Alexander Assmann

**Affiliations:** 1 Department of Cardiovascular Surgery, Heinrich Heine University, Medical Faculty, Duesseldorf, Moorenstrasse 5, Germany

## Background/Introduction

Post-sternotomy pain often causes patients discomfort and delays recovery. Consecutive pulmonary complications, in particular infections, as well as adverse effects of pain killers and addiction to pain killers lead to a significant increase of postoperative complication rates.

## Aims/Objectives

In order to prevent postoperative pain and subsequently impaired inspiration enhancing the risk of pulmonary complications, sufficient analgesia is essential in patients after sternotomy. The present prospective randomized pilot study aimed at investigating the influence of Kinesio taping on the post-sternotomy pain levels.

## Method

Fifty elective cardiosurgical patients at the age of 66 ± 10 years undergoing sternotomy between 09/2014 and 11/2014 at the University Hospital Duesseldorf were enrolled in the trial. All patients were randomly assigned to either group TAPE (n = 25), receiving Kinesio taping after discharge from the intensive care unit, or group CONTROL (n = 25) without any taping. In each group, two drop-outs occurred. Until discharge from the hospital, the pain level of all patients was daily assessed using a standardized pain measurement scale ranging from 0 to 10 points. Moreover, the need for additional analgesic therapy was documented.

## Results

The mean hospitalization period in group TAPE was 10.1 ± 2.2 days versus 11.4 ± 4.4 days in group CONTROL. Patients treated with Kinesio tapes had significantly lower pain scale levels as compared to controls (1.7 ± 0.7 versus 3.3 ± 1.7 with p < 0.0001). The dose of administered opioids (piritramide) as well as of non-opioid analgesics (paracetamol) were significantly reduced in group TAPE (0.7 ± 0.9 versus 1.3 ± 1.1 mg/day and 0.9 ± 0.6 versus 1.3 ± 0.6 g/day; both with p < 0.05). The percentage of patients presenting with unimpaired breathing amounted to 96% in group TAPE and 22% in group CONTROL (p < 0.0001).

## Discussion/Conclusion

Kinesio taping seems to be a safe and promising approach to reduce postoperative pain and the dose of analgesic drugs to be administered after cardiac surgery via sternotomy. The results of this pilot study warrant a clinical trial with a higher number of participants.

